# Prevalence of Maternal Third Delays in Emergency Obstetric Care and Determinant Factors Among Women Giving Birth in Government Hospitals in East Gojjam Zone, Northwest Ethiopia: A Cross‐Sectional Study

**DOI:** 10.1002/hsr2.72384

**Published:** 2026-04-16

**Authors:** Habtamu Wudu, Werkneh Minalu

**Affiliations:** ^1^ Department of Statistics (MSc in Biostatistics), College of Natural and Computational Science Gambella University Gambella Ethiopia; ^2^ Departments of Public Health (MPh in Reproductive health) College of Health Sciences, Tropical College Debre Markos Ethiopia

**Keywords:** emergency obstetric care, Ethiopia, hospital clustering, maternal third delay, multilevel logistic regression

## Abstract

**Background:**

The third delay involves any delay in seeking or receiving appropriate emergency obstetric care after presenting at a health facility. It is the most contributing factor for maternal death in developing countries. Limited attention has been paid to maternal third delay and determinant factors at public hospitals, East Gojjam zone, Ethiopia.

**Objective:**

The aim of this study was to assess the Prevalence of maternal third delay during emergency obstetric care and Determinant factors among women who gave birth in government hospitals in East Gojjam Zone, Northwest Ethiopia.

**Method:**

An institution‐based cross‐sectional study was implemented among 525 women (using systematic sampling) from September 01 – December 30, 2023. Data were collected using a structured questionnaire presented by an interviewer and extracted from observational checklists. The collected data were coded and entered using EpiData version 3.1, and the Chi‐squared test of association and multilevel logistic regression were done using STATA version 14 statistical software. *p* value < 0.05 was declared statistically significant.

**Results:**

This study indicates that 32.2% [95% CI: 28.3–35.9%] of women were third delayed. Additionally, time of admission AOR = 0.46 [95% CI: 0.23–0.98], ANC follow‐up AOR = 1.98 [95% CI:1.32–3.93], absence of health professionals AOR = 2.85 [95% CI: 1.98–4.35], types of pregnancy (Wanted but unplanned) AOR = 1.49 [95% CI: 1.31–3.54), Unwanted and unplanned AOR = 2.98 [95%CI:2.51–4.69], and lack of medical supply/medication AOR = 1.56 [95%CI: 1.34–3.67] were found to be significant determinant factors of maternal third delay.

**Conclusion:**

The magnitude of the third delay was significantly high. Driven by factors like delayed admission, inadequate ANC, and resource shortages. Strengthen maternal healthcare, improve timely access, and address resource and planning gaps for better outcomes. These interventions are crucial to reduce maternal third delays.

**Abbreviations**
ANC: antenatal care
AOR: adjusted odds ratio
CBHI: community‐based health insurance
CI: confidence interval
COR: Crude odds ratio
CSH: comprehensive specialized hospital
df: degree of freedom
EMOC: emergency obstetrics care
MMR: maternal mortality rate
PH: primary hospital
SD: standard deviation
WHO: World Health Organization
*χ*²: Chi‐squared

## 1 Introduction

The maternal third delay has been conceived within the three delays model published by Thaddeus and Maine in 1994. This model describes the third delay as the delay in receiving quality and appropriate emergency obstetric care after the woman has reached a health facility. While The first delay occurs when deciding to seek care, and the second delay involves locating and accessing the health facility [1]. The first two delays are related to demand‐side barriers that prevent women from utilizing and accessing delivery services [2]. The third delay in accessing maternal health care is closely related to several factors concerning health facilities and the quality of care offered. These factors include a lack of emergency obstetric care services and medication supplies, a shortage of trained staff, ineffective management of emergency care, prolonged waiting times, inadequate referral practices, and poor coordination among staff, drugs and equipment, rules and regulations, personnel, facility infrastructure, patient and referral‐related issues [3, 4].

In developing countries, many maternal deaths and adverse outcomes still happen after mothers have already arrived at health facilities; the urgency for quality obstetric care is understandable [4, 5, 6, 7]. Consequently, the focus has shifted to delays within health facilities, rather than delays in deciding to seek care, as the major contributor to maternal mortality [8]. The current global maternal mortality ratio (MMR) stands at 197 deaths per 100,000 live births, which is unacceptably high. Alarmingly, 94% of these deaths occur in low‐ and middle‐income countries [9]. Ethiopia has the most elevated MMR of 267 per 100,000 live births among the 16 Sub‐Saharan African countries. The differences in MMR between low and high‐income countries arise from differences in health care service utilization and in the quality of care provided [9, 10].

This is two‐thirds of the odds in Ethiopia. For instance, more than one third of mothers in Ethiopia have been experienced third delay [11, 12, 13] and in the national Maternal Death Surveillance Report, third delay caused 36.3% of the total number of maternal death [14] which emphasizes the importance of health facility issue affecting not only the outcome of the patient but also the subsequent choice by the community to attend emergency obstetric care.

Timely access to Emergency Obstetric and Newborn Care (EmONC) services is crucial for reducing maternal and neonatal mortality in low‐income countries such as Ethiopia. This evidence‐based service is vital for managing potentially life‐threatening complications that can affect women and newborns during pregnancy, childbirth, and the immediate postpartum period [15]. The proper implementation of Emergency Obstetric and Newborn Care (EmONC) can significantly reduce maternal and perinatal deaths and complications [16, 17, 18]. However, in Ethiopia, EmONC services remain limited and in accessible to many women due to inadequate infrastructure, insufficient resources, and a shortage of trained personnel [19].

The World Health Organization (WHO) addresses the maternal third delay through its Ending Preventable Maternal Mortality (EPMM) strategy, which emphasizes universal health coverage, tackling inequities, and improving health systems and care quality. In addition to this, The National Minister of Health's (MOH) agencies in Ethiopia, implement specific strategies for each delay, including community‐based interventions, financial risk pooling for care access, maternity waiting homes, free transportation to care, enhancing facilities for adequate emergency obstetric and newborn care (CEmONC), and utilizing maternal death surveillance and response (MDSR) [14, 23]. Despite these efforts to reduce maternal third delay in emergency obstetric care remains poorly understood and inadequately studied, particularly in resource‐limited settings. Maternal third delay is still a common problem.

This study is the first multilevel analysis of the prevalence and determinants of maternal third delay in East Gojjam, explicitly accounting for both individual‐ and facility‐level influences on third delay. Its exclusive focus on women delivering in hospitals contrasts with prior community‐based or mixed‐facility studies [13, 20, 21]. Many studies were conducted and showed that the absence of health professionals, being self‐employed, governmental employed, having ANC follow‐up [20], complications during labor, level of health institution, inadequate infrastructure and supplies, including bed and drug supplies, insufficient health personnel [21, 22] were determinant factors in maternal third delay. In Ethiopia, previous studies show that the prevalence of maternal third delay was 74.7% in Addis Ababa, 29.3% in Sidama regional state, and 34.8% in Gurage zone; they used single‐level approaches [13, 20, 21]. By restricting the setting to hospitals only, the study is differentiated from community‐based work and better isolates how hospital‐level factors shape the third delay. Together, the multilevel analysis and hospital‐only focus provide more granular evidence than earlier studies for targeting quality‐improvement efforts for quality of obstetric services in East Gojjam Zone. Therefore, this study aimed to assess the prevalence of maternal third delay during emergency obstetric care and its determinant factors among women who give birth at governmental hospitals in East Gojjamm Zone, Northwest Ethiopia.

## 2 Methods

### 2.1 Study Design and Setting

An institution‐based cross‐sectional study design was employed at governmental hospitals in East Gojjam zone, Northwest Ethiopia. Geographically, it extends from 9° 55’ 01” to 11° 14’ 12” north and from 37° 29’ 37” to 38°30’ 18'east. As the zonal health office report in 2020 showed, the East Gojjam zone has a total population of 2,719,118 and 632,353 households. It also has 423 health posts, 102 health centers, 9 primary hospitals, one general hospital, and one comprehensive specialized hospital [24]. The study was conducted from September 1 to December 30, 2023, among mothers who gave birth at governmental hospitals.

### 2.2 Study Population

All women who gave birth at governmental hospitals in the East Gojjam zone were the source population. All women who gave birth at the selected governmental hospitals in East Gojjam zone during the study period were the study population.

### 2.3 Eligibility and Exclusion Criteria

All women who were admitted for emergency obstetric care in the selected Governmental hospitals and those Women who volunteered to participate in the study are included in the study. Women who were readmitted after an earlier inclusion and those who were transferred to another facility after earlier inclusion were excluded. Additionally, women who were critically ill and unable to communicate during the data collection period were excluded from the study.

### 2.4 Sample Size Determination and Sampling Procedure

#### 2.4.1 Sample Size Determination

The sample size was calculated using the formula for a single population proportion, based on a third delay proportion (*P* = 29.3%) from a previous study, 95% confidence interval (CI), and the margin of error (d) of 0.05. To account for clustering of women within hospitals, a design effect of 1.5% was applied, which was based on an anticipated intra‐class correlation coefficient (ICC) derived from similar maternal health studies that reported non‐negligible within‐facility correlation of outcomes [20]. After adding 10% nonresponse rate, the final required sample size was 528.

#### 2.4.2 Sampling Techniques and Procedures

To choose participants from the study population, a multistage sampling procedure was employed. First, using simple random sampling, select 4 governmental primary Hospital (lumame primary hospital, Dejen primary hospital, Yejube primary hospital and Bichena primary hospital) from 9 governmental primary Hospitals (Dejen primary hospital, Lumame primary hospital, Bichena primary hospital Debere work primary hospital, Bibugn primary hospital, Yejube primary hospital, Debre Elias primary hospital, Mertulemariam primary hospital and Shebel Berenta primary hospital), Mota general hospital and Debre Markos comprehensive specialized hospital were taken directly since there was no other general and comprehensive specialized hospital in the study area. The baseline number of women ($N_{j}$) expected to be admitted for emergency obstetric care in each selected hospital during the study period was obtained from the previous year's total deliveries, and the overall total (N) across the six hospitals was 1,420 women. The total sample size (528) was proportionally allocated to 6 selected Governmental Hospitals using the following proportional Allocation formula. Using the proportional Allocation formula

$$n_{j}=\frac{n*N_{j}}{N},\;j=1,2,\ldots 6$$

**Figure 1:**
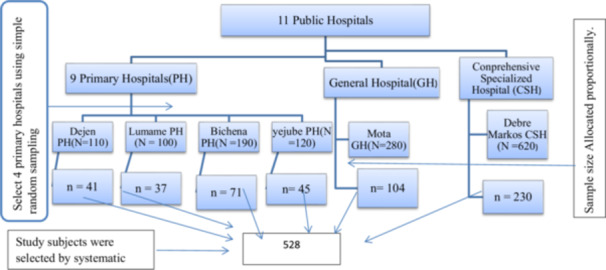
Schematic presentation showing the sampling procedure to select the number of participants from each hospital in the East Gojjam zone.

Where *n* is the total sample size (n = 528), nj is the sample size for each selected hospital in the study area, N is the total number of prior‐year deliveries across the six hospitals, and Nj is the prior‐year number of deliveries in hospital (j) (Figure 1).

Study participants were selected from the study population by systematic random sampling (SRS), in which samples were drawn every *K* of admitted Mother. After calculating *K* = *N*/*n*, where “*N*” is the total number of women expected to be discharged from the hospital after they got emergency obstetric care in the study area during the study period based on the previous year statistics and “*n*” is the calculated sample size; study participants were selected at every K^th^ interval (1420/528 = 2.69), Which was rounded to 3, so every 3rd eligible woman was selected after a randomly chosen first participant by lottery. Of the 528 women approached, 525 participated, yielding a 99.4% response rate (525/528). Three women were excluded from the final analysis because they met the predefined exclusion criteria (readmission, transfer to another facility after earlier inclusion, or being critically ill and unable to communicate during data collection).

##### 2.4.2.1 Variables of the Study

###### Dependent Variable

Maternal third delay during emergency obstetric care and it is dichotomized as 1 presence (third delayed/yes) and 0 being not present (not third delayed/no).

###### Independent Variables

Socio‐demographic and economic factors, obstetric and individual factors, and Health Facility‐Related factors.

### 2.5 Operational Definition

#### 2.5.1 Third Maternal Delay

the time between arriving at the health facility and receiving the initiation of appropriate care services. A delay is considered present when there is an unjustified waiting time of ≥ 1 h before receiving any form of maternal care intervention (such as assessment, monitoring, counseling, or initiation of delivery management) [29].

#### 2.5.2 Emergency Obstetric Care

A care given to a pregnant, laboring, or postpartum woman with emergency medical, surgical, or obstetrical problems.

#### 2.5.3 Knowledge on Key Danger Signs of Labor and Childbirth

When a woman spontaneously mentioned at least three major risk indications for labor and delivery, it was assumed that she was knowledgeable about these signs [26].

#### 2.5.4 Admitted for Emergency Obstetric Care

A woman was considered as she was admitted for emergency obstetric care when she was admitted during her pregnancy, labor and delivery, or postpartum period for emergency obstetric care which includes assisted vaginal delivery; removal of retained products of conception; manual removal of the placenta; parenteral administration of oxytocin, antibiotics, and anticonvulsants; blood transfusion; and cesarean section (surgery) [27, 28].

#### 2.5.5 Absence of Health Professionals

A woman was classified as having experienced an absence of health professionals if, at any time during her current hospital admission, she needed care (e.g., for pain, bleeding, or labor assessment) but reported that no midwife, nurse, or doctor was available to attend to her at that moment. This variable was measured using a yes/no question at discharge (“During this admission, was there any time when you needed a health professional but none was available?”) and therefore captures the occurrence of at least one such episode, not the percentage of total admission time without staff [29].

##### 2.5.5.1 Lengthy Admission Process

The time span of the complete admission chain is determined by the amount of time it takes (measured in minutes) from when a mother arrives at the hospital until she is placed in a bed or assigned to a ward (the point when the mother starts receiving any obstetric care she may need, whether i.e. being examined or beginning any therapies). A mother who takes 30 min or more to go through the complete admission process is considered to have had a lengthy admission process [25].

### 2.6 Data Collection Tools and Procedures

The data collection tools were adopted and modified after reviewing various literature sources [29, 30]. The data were collected by using face‐to‐face interviewer‐administered and pre‐tested, structured questionnaires and extracted from a medical observational checklist. The referral paper, triage paper, and patient chart were reviewed to assess the woman's condition. A structured questionnaire was administered daily in the inpatient wards at the time of discharge to gather information about her demographic characteristics, any reasons for delays, and her experiences in the hospital.

### 2.7 Data Quality Assurance

Initially, it was prepared in English. The English version of the questionnaire was translated into the local language (Amharic) and back‐translated into English by language experts to check for its original meaning. The investigators conducted a 3‐day training session for data collectors and supervisors, covering the study objectives, data collection tools and procedures, confidentiality, and informed consent. Six obstetric nurses fluent in Amharic from each hospital were recruited to assist with data collection. Throughout the data collection process, six supervisors for each Hospital closely monitored, checked, and reviewed the data daily to ensure clarity, consistency, and completeness.

### 2.8 Data Processing and Analysis

The collected data were entered into EpiData version 3.1; the data were exported for analysis into STATA version 14. Descriptive statistics were computed, and results were presented using tables and narration. Chi‐squared (*χ*²) test of association was conducted to identify factors that had a significant association with maternal third delay using a 5% level of significance. Both binary and multivariable analyses were carried out to identify determinant factors on the prevalence of maternal third delay. Variables with *p*‐value < 0.25 in binary analysis were considered as candidates for the final model. A multilevel logistic regression analysis was used to identify determinant factors of the third delay. In multilevel logistic regression, adjusted odds ratio (AOR) with a 95% confidence interval (CI), test of heterogeneity of proportion between hospitals, interclass correlation coefficient (ICC), and model comparison were assessed. A priori significance level was set at *α* = 0.05. All statistical tests were two‐sided.

### 2.9 Ethics Approval and Informed Consent of Participants

This study follows all principles outlined in the Declaration of Helsinki, and ethical approval to carry out this study was sought from an Institutional Review Board (IRB) from the College of Health Science and Medicine, Debre Markos University, Ethiopia, with reference number IRB/CHSM/324/153. Additionally, permission was also sought from the Health Bureau in East Gojjam, and this took place before data collection from September 1 to December 30, 2023, in governmental hospitals in East Gojjam, Northwest Ethiopia. As a vital part of human subjects in compliance with the Declaration of Helsinki, free and informed consent was obtained from all participating individuals before conducting the study. The IRB approved the verbal informed consent approach, as there was minimal risk, which was applicable because the data collection approach was carried out through an interview technique. Participants were fully informed of the intention of this study, including the procedure, sources of funding, as well as the right to decline participation or withdraw from the study at any time without any form of retaliatory measures. All data obtained from the participating subjects was kept confidential. Additionally, anonymity was maintained throughout the study.

## 3 Results

### 3.1 Socio‐Demographic and Economic Characteristics of the Respondents

From a total of 528 women, 525 women were included in the study during the study period, yielding a 99.4% response rate. Of these, 371 (70.7%) of the respondents were aged between 21 and 34 years old, and The mean age of the participating mothers was 27 years, with a standard deviation (SD) of 3.6 years. Most of the respondents, 283 (53.9%), were housewives, and 317 (60.4%) of respondents were urban dwellers. Regarding the educational status of women, 86 (16.3%) have no formal education, 169 (32.2%) (Table 1).

**Table 1:** Socio‐demographic characteristics of mothers in East Gojjam Zone, Northwest Ethiopia, 2023.

Variable	Categories	Frequency (%)
Age in years	15–20	97 (18.5)
	21–34	371 (70.7)
	≥ 35	57 (10.8)
Religion	Orthodox	282 (53.7)
	Muslim	159 (30.2)
	Others*	84 (16.1)
Marital status	Married	402 (76.6)
	Single	45 (8.6)
	Others**	78 (14.8)
Educational status of women	No formal education	86 (16.3)
	Primary (1–8)	169 (32.2)
	Secondary	215 (40.9)
	College and above	55 (10.5)
Occupational status of women	Housewife	283 (53.9)
	Self‐employee	123 (23.4)
	Government employee	75 (14.3)
	Others***	44 (8.4)
Residence	Urban	317 (60.4)
	Rural	208 (39.6)
Family monthly income	≤500ETB	159 (30.5)
	501–1000ETB	238 (45.3)
	1001–2000 ETB	81 (15.4)
	≥2001ETB	47 (8.8)
Community‐based health insurance	Yes	335 (63.8)
	No	190 (36.2)
Age (mean)		27 ± 3.6 y

* Protestant and Catholic.

** Divorced and widowed.

*** Private employee and student. Y, years.

### 3.2 Health Facility‐Related Characteristics of the Study Participant

From the total participants in the selected hospital, 314 (59.8%) of mothers live in a physical distance greater than 5 km to reach nearby hospitals, 345 (65.7%) of the respondents arrived at the hospital during the daytime, and 328 (62.5%) were during working days. Concerning the absence of health professionals, 181(34.5%) respondents reported experiencing an absence of health professionals during their current hospital admission (Table 2).

**Table 2:** Health facility‐related characteristics of study participants in East Gojjam Zone, Northwest Ethiopia, 2023.

Variable	Categories	Frequency (%)
Road unavailability	Yes	303 (57.6)
	No	222 (42.3)
Distance from the hospital	≤ 5 km	211 (40.2)
	> 5 km	314 (59.8)
Transportation unavailability	Yes	195 (37.1)
	No	330 (62.9)
Absence of a health professional	Yes	181 (34.5)
	No	344 (65.5)
Lengthy admission process	Yes	95 (18.1)
	No	430 (81.9)
Time of admission	Day	345 (65.7)
	Night	180 (34.3)
Date of admission	Weekend/Holiday	197 (37.5)
	Work day	328 (62.5)
Lack of medical supplies/medication	Yes	150 (28.6)
	No	375 (71.4)
Referral case	Yes	107 (20.4)
	No	418 (79.6)

### 3.3 Obstetric Related Characteristics of the Study Participant

A total of 78 (14.9%) had a history of abortion, and 477 (85.1%) of them had no history of obstetric complications. From the study participants, 324 (61.7%) had good knowledge of danger signs of labor/childbirth, the majority of women delivered spontaneously (90.1%), and no cesarean sections were recorded. This may be due to the specific study period and exclusion of women who were readmitted, transferred to other facilities, or critically ill and unable to communicate during data collection (Table 3).

**Table 3:** The obstetric‐related characteristics of study participants, East Gojjam Zone, Northwest Ethiopia, 2023.

Variables	Categories	Frequency (%)
ANC follow‐up	Yes	427 (81.3)
	No	98 (18.7)
History of abortion	Yes	78 (14.9)
	No	447 (85.1)
Type of pregnancy	Wanted and planned	361 (68.8)
	Wanted but unplanned	108 (20.6)
	Unwanted and unplanned	56 (10.6)
History of obstetric complication	Yes	78 (14.9)
	No	447 (85.1)
Mode of delivery	Spontaneous vaginal delivery	473 (90.1)
	Instrumental delivery	52 (9.9)
Knowledge of danger signs	Poor	201 (38.3)
	Good	324 (61.7)

### 3.4 Magnitude of Third Maternal Delays

**Figure 2:**
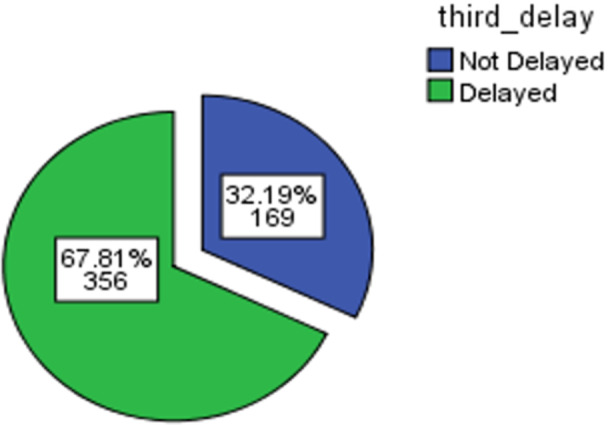
magnitude of third maternal delay of study participants in East Gojjam zone, Northwest Ethiopia, 2023.

This finding indicates that from a total of 528 mothers, 525 study participants were included in the study area and time, 169 (32.2%) with [95% CI; 28.3–35:9] of the participants had maternal third delay. Women were waiting for more than an hour to receive maternal care service (Figure 2).

### 3.5 Factors Associated with Third Maternal Delay

#### 3.5.1 Chi‐Square Test of Association

As we have discussed in the methodology section, we select the factors that were statistically associated with the maternal third delay. This study shows that Age, Absence of health professional, Lengthy admission process, Time of admission, Lack of medical supply/medication, ANC follow‐up, Type of pregnancy, History of obstetric complication, Mode of delivery, and mothers' knowledge on danger signs were significantly associated with third delay at 5% level of significance (Table 4).

**Table 4:** Chi‐ squared test of association between sociodemographic, health facility‐related, obstetric‐related characteristics, and maternal third delay.

Independent variables	Categories	Maternal third delay		Chi‐square (χ2) (df)	*p*‐value	COR[95%CI]
		Yes	No			
Age in years	15–20^@^	11	86	28.295 (2)	*p* value < 0.001	
	21–34	144	227			0.34 [0.25–0.93]
	≥ 35	14	43			1.23 [1.13–2.84]
Marital status	Married@	133	269	1.167 (2)	0.71	
	Single	15	30			2.34 [0.56–2.98]
	Others**	21	57			2.45 [0.96–3.38]
Educational status of women	No formal education@	29	57	3.018 (3)	0.39	
	Primary (1–8)	50	119			0.62 [0.32–2.45]
	Secondary	67	148			3.23 [0.55–4.56]
	College and above	23	32			1.78 [0.99–2.65]
Occupational status of women	Housewife@	83	200	6.640 (3)	0.08	
	Self‐employee	43	80			1.89 [0.89–2.44]
	Government employee	22	53			2.33 [0.66–3.18]
	Others***	21	23			2.10 [0.76–2.98]
Type of pregnancy	Wanted and planed^@^	93	268	27.883 (2)	*p* value < 0.001	
	Wanted but unplanned	57	51			1.32 [1.21–4.54]
	Unwanted and unplanned	19	37			2.43 [1.56–4.78]
Residence	Urban@	112	205	3.616 (1)	0.06	
	Rural	57	151			1.56 [0.76–2.75]
Family monthly income	≤ 500ETB@	50	109	0.250 (3)	*p* value > 0.99	
	501–1000ETB	78	160			0.54 [0.26–2.18]
	1001–2000 ETB	27	54			1.54 [0.96–3.18]
	≥ 2001ETB	14	33			0.78 [0.42–3.22]
Community‐based health insurance	No@	60	130	0.017 (1)	*p* value > 0.99	
	Yes	109	226			3.23 [0.98–2.23]
Lengthy admission process	No@	109	321	49.240 (1)	p value < 0,001	
	Yes	60	35			1.84 [1.28–3.23]
Road unavailability	No@	64	158	1.733 (1)	0.19	
	Yes	105	198			0.35 [0.15–1.45]
Distance from the hospital	≤ 5 km@	62	149	1.067 (1)	0.30	
	> 5 km	107	207			2.45 [0.75–5.45]
Transportation unavailability	No@	112	218	1.039 (1)	0.30	
	Yes	57	138			0.56 [0.25–1.35]
Absence of a health professional	No^@^	146	198	46.686 (1)	0.02	
	Yes	23	158			1.86 [1.34–4.35]
Time of admission	Night@	25	155	40.765 (1)	*p* value < 0.001	
	Day	144	201			0.57 [0.24–0.99]
Date of Admission	Weekend/Holiday@	67	130	0.354 (1)	*p* value > 0.99	
	Work day	102	226			0.44 [0.13–1.56]
Lack of medical supplies/medication	No@	154	221	45.961 (1)	*p* value < 0.001	
	Yes	15	135			1.34 [1.23–3.56]
Referral case	No@	143	275	3.393	0.06	
	Yes	26	81			0.78 [0.54–2.23]
ANC follow‐up	No	17	81	11.341	*p* value < 0,001	1.75 [1.34–3.78]
	Yes@	152	275			
History of abortion	No@	148	299	0.898 (1)	0.34	
	Yes	21	57			2.34 0.98–3.23]
History of obstetric complication	No@	158	289	12.775 (1)	*p* value < 0.001	
	Yes	11	67			1.23 [1.59–3.55]
Mode of delivery	spontaneous vaginal delivery@	135	338	27.471 (2)	*p* value < 0.001	
	Instrumental delivery	34	18			1.25 [1.23–3.93]
Religion	Orthodox@	97	185	2.055 (2)	0.58	
	Muslim	50	109			0.78 [0.45–2.31]
	Others*	22	62			1.25 [1.12–3.23]
knowledge of danger signs	Poor@	43	158	16.603 (1)	*p* value < 0.001	
	Good	126	198			1.14 [1.03–2.35]

* Protestant and Catholic.

** Divorced and widowed.

*** Private employee and student. df: degree of freedom.@ Reference group

#### 3.5.2 Multivariable Logistic Regression Analysis

From the bivariable analysis, Age, Type of pregnancy, Lengthy admission process, Time of admission, lack of medication/supply, ANC follow up, history of obstetric complication, mode of delivery, knowledge of danger signs, and Absence of health professional were statistically significant at the 0.25 level of significance.

A chi‐square test statistic was applied to assess the test of heterogeneity in the proportion of women who delayed receiving obstetric care after arriving at the 6 hospitals. The test yield *χ* ^2^ = 195.804, df = 5, *p* < 0.001. It indicates that maternal third delays vary significantly among the hospitals. It is also supported by the variance of the random effect ($\mathrm{Var}\left(u_{0j}\right)$) = 0.513 (*p* = 0.002). Therefore, we conclude that there is significant variation in maternal third delay. A multilevel logistic regression is appropriate to model the binary outcome of delay, accounting for clustering within hospitals and exploring both patient‐level and hospital‐level predictors.

Model comparison for the multilevel logistic regression was conducted using the Akaike Information Criterion (AIC) and the Bayesian Information Criterion (BIC), and overall model adequacy was assessed using a deviance‐based chi‐square test. The AIC and BIC values for the random‐intercept model were 2335.71 and 2367.23, respectively, whereas the corresponding values for the empty (null) model were 2943.21 and 2857.43. These lower AIC and BIC values indicate that the random‐intercept multilevel model provides a better fit for predicting the prevalence of third delay across hospitals than the empty model and alternative specifications. In addition, the deviance‐based chi‐square test was statistically significant (*χ*² = 102.5, *p* < 0.001), supporting that the random‐intercept model adequately fits the data. The intra‐hospital correlation coefficient (rho) or variance partition coefficient is a measure of variation of prevalence of third maternal delay by hospital (ICC/VPC = 0.135), meaning that 13.5% of variation in the prevalence of third delay can be explained by grouping (hospitals). The remaining 86.5% of the variation is explained within the hospital.

According to the result of the random intercept model, absence of health professional, ANC follow up, types of pregnancy, time of admission, and Lack of medical supply/medication were found to be significant factors of variation in the prevalence of third delay among hospitals (Table 5).

**Table 5:** Results of random intercept multilevel logistic analysis of women admitted to emergency obstetric care, East Gogjiam Zone, Northwest Ethiopia, 2023.

Variables	Fixed effect covariates		COR [95%CI]	AOR [95% CI]	
	Maternal third delay				
	Yes *n* (%)	No *n* (%)			
Age					
15–20^@^	11 (11.3)	86 (88.7)			
21–34	144 (38.8)	227 (61.2)	0.34 [0.25–0.93]	0.77 [0.35–1.73]	
≥ 35	14 (24.6)	43 (75.4)	1.23 [1.13–2.84]	0.99 [0.34–3.45]	
Absence of a health professional					
No^@^	139 (40.4)	205 (59.6)			
Yes	30 (16.6)	151 (83.4)	1.86 [1.34‐4.35]	2.85 [1.98‐4.35]**	
History of obstetric complication					
No^@^	158 (35.3)	289 (64.7)			
Yes	11 (14.1)	67 (85.9)	1.23 [1.59–3.55]	1.21 [0.89–3.24]	
Lack of medical supplies/medication					
No^@^	154 (41)	221 (59)			
Yes	15 (10)	135 (90)	1.34 [1.23–3.56]	1.56 [1.34–3.67]**	
ANC follow‐up					
Yes@	152 (36)	275 (64)			
No	17 (17.3)	81 (82.7)	1.75 [1.34–3.78]	1.98 [1.32–3.93]*	
Time of admission					
Night @	25 (13.8)	155 (86.2)			
Day	144 (41.7)	201 (58.3)	0.57 [0.24–0.99]	0.46 [0.23–0.98]*	
Lengthy admission Process					
No^@^	109 (25.3)	321 (74.7)			
Yes	60 (63.2)	35 (36.8)	1.84 [1.28–3.23]	1.39 [0.91–2.93]	
Mother's knowledge of danger signs					
Poor ^@^	43 (21.4)	158 (78.6)			
Good	126 (38.9)	198 (61.1)	1.14 [1.03–2.35]	0.58 [0.23–0.94]	
Types of pregnancy					
Wanted and planned@	93 (25.8)	268 (74.2)			
Wanted but unplanned	57 (52.7)	51 (47.2)	1.32 [1.21–4.54]	1.49 (1.31–3.54)*	
Unwanted and unplanned	19 (33.9)	37 (66.1)	2.43 [1.56–4.78]	2.98 [2.51–4.69]*	
Mode of delivery					
Spontaneous vaginal delivery@	135 (28.5)	338 (71.5)			
Instrumental delivery	34 (65.4)	18 (34.6)	1.25 [1.23–3.93]	1.23 [0.35–4.32]	
**Random Part**		**Estimate**	**S.E**	***z***	***p*‐value**
Level‐two variance(hospital) $\left({\sigma_{0}}^{2}=\mathrm{Var}\left(u_{0j}\right)\right)$		0.513	0.221	2.041	0.002
**Model Selection Criteria and Model Adequacy Test**					
AIC	BIC	Deviance‐based chi‐square test(ˆ²)	ICC(VPC)		
2335.71	2367.23		102.5 (*p* < 0.001)	ICC(VPC) = $\frac{{\sigma_{0}}^{2}}{{\sigma_{0}}^{2}+\pi ²/3}=0.135$	

*Note:* @ = reference group.

Abbreviations: AOR, adjusted odds ratio; COR, crude odds ratio; ICC, intra‐class correlation; VPC, Variance Partition Coefficient.

* = significant at *p* value < 0.05,

** =significant at *p* value < 0.001.

Women who have no ANC follow‐up were two times more likely, AOR = 1.98 [95%CI = 1.32–3.93], more likely to face maternal third delay than those who have ANC follow‐up. One potential explanation for this relationship is that when women receive ANC follow‐up, it provides them with greater preparedness, familiarity with hospital procedures, and establishes continuity of care through documentation. This, in turn, sets up a smoother path for women to be admitted to a hospital and begin receiving services much quicker than those who did not receive ANC follow‐up. In contrast, women who do not receive ANC follow‐up will typically arrive at a hospital with no pre‐existing records or identification of risk factors, which requires additional assessments and additional administrative steps prior to being admitted to a hospital. This additional administrative burden results in delays of admission and contributes to the third delay.

In this study, women who were unwanted and unplanned pregnant had approximately three times, AOR = 2. 98 [95% CI = 2.51–4.69] more likely to face maternal third delay compared to women who were wanted and planned pregnant. This relationship may be related to the fact that pregnant women who are experiencing unwanted pregnancies, on average, have less access to antenatal care and less contact with healthcare services during their pregnancy. When a woman arrives at a hospital with an unwanted pregnancy, she is likely to require additional evaluations, counseling, or administrative steps to be registered as a non‐newborn for treatment by that facility; all of these activities add time to her admission process and increase the likelihood of delays. Women who wanted and were unplanned pregnant had 1.5 times (AOR = 1.49 [95% CI = 1.31–3.54] more likely to face maternal third delay compared to women who were wanted and planned pregnant. Women who were admitted to emergency obstetric at day were approximately 0.54 times less likely to face third maternal delay compared to at night.

## 4 Discussion

This institution‐based cross‐sectional study has attempted to identify the magnitude (prevalence) and associated factors of maternal third delay in governmental hospitals in East Gojjam zone, Northwest Ethiopia. A multilevel random intercept multilevel logistic regression model showed that the prevalence of maternal third delay varies across hospitals, and it assumed that the effects of explanatory variables are the same for each hospital.

The prevalence of maternal third delay among women admitted for emergency obstetric care in governmental hospitals of East Gojjam Zone, Northwest Ethiopia, was found to be 32.2% [95% CI: 28.3–35.9]. This finding is consistent with studies conducted in Sidama Regional State (29.3%) [20]. In Gamo Zone (31.7% [29], Yem Special Woreda (34.7%), and Gurage Zone (34.8%) [12, 21]. However, it was higher than that reported in Mozambique (14.2%) [4]. The discrepancy may be explained by methodological differences, as the Mozambique study utilized secondary data and assessed only delays associated with maternal deaths, excluding mothers who survived but experienced delays in accessing maternity care. Conversely, the prevalence observed in the present study was lower than that reported in Addis Ababa (74.7%), Nigeria (57%), and Pakistan (71%) [13, 31, 32]. This variation could be attributed to differences in study settings and additional factors such as population differences, study scope, methodological variations, absence of health extension programs, and lifestyle changes may also contribute to the observed discrepancies.

In this finding, different variables, like time of admission, ANC follow‐up, absence of health professionals, types of pregnancy, and Lack of medical supply/medication, were significant predictors of maternal third delay. When we see other studies conducted, like in Sidama regional state, women's occupational status, referral case, ANC follow‐up, and absence of health professionals were significant predictors of maternal third delay [20].

Women who had no antenatal follow‐up were approximately two times more likely to experience a third delay compared to those who had one or more antenatal follow‐ups. This suggests that no‐ANC status creates a strong causal pathway through birth preparedness deficits that ultimately slows facility‐level care. A similar result was also found, consistent with a study done in the Arsi Zone and Sidama regional state [20, 33]. Furthermore, a study carried out in West Bengal, India, revealed that a larger percentage of maternal third delays were experienced by women who did not attend ANC [34]. This could be because women who had ANC follow‐up are likely to be aware of the warning signals of pregnancy and to be well‐prepared for labor and any complications that may arise [14]. Other causes could include women's communication experiences with healthcare professionals; they may know who to talk to and how to talk about issues. When comparing prevalence thoughtfully, our results align with existing literature. Our observed AOR of 1.98 is slightly more conservative than the pooled estimate of 2.90 [95% CI: 1.30–4.10 reported in recent Ethiopian meta‐analyses [35]; it remains consistent in direction and magnitude with regional studies in South Gondar (AOR ≈ 1.8) [36]. This consistency underscores that even a moderate lack of ANC nearly doubles the risk of care‐seeking delays due to inadequate preparation.

Concerning the lack of a health professional, Women who said they didn't receive the care they needed from medical experts were three times as likely to have a maternal third delay in comparison to their rivals. This outcome is consistent with research conducted in Ethiopia's Sidama regional state and in the Gamo Zone. Which identified that women who reported the absence of health professionals when they needed them were ten and five times more likely to experience maternal third delay [20, 29]. This could be because of staff workload and/or motivation, which could result in a gap in the availability of health professionals to provide care.

The lack of medical supplies or medication was another factor related to a third parental delay in using institutional delivery services. According to this study, women who weren't necessary to take medicine had a higher chance of postponing their use of institutional delivery services. The systematic journal finding conducted in Ethiopia's Gamo Zone and developing nations corroborated this conclusion [14, 29].

Time of Admission, it is also a significant factor in the third maternal delay of women's emergency obstetric care. This study shows that Women who were admitted to emergency obstetric at day were approximately decrease by 54% times less likely to face third maternal delay compared to at night. This may be due to the availability of health professionals and other health facilities during day time. This factor is insignificant in different studies conducted in a systematic review study, Sidama regional state, and Gamo zone [14, 20, 29].

The last factor that has a significant effect on third maternal delay of women's emergency obstetric care was types of pregnancy, women who were unwanted and unplanned pregnant had more likely to face maternal third delay compared to women's who were wanted and planned pregnant, and women who were wanted and unplanned pregnant had 1.5 times more likely to face maternal third delay compared to women's who were wanted and planned pregnant.

This Study has some limitations; first, due to the fact that it is cross‐sectional, causation cannot be determined from either data set. Second, the information collected via both interviewers' questionnaires and during discharge interviews is subject to recall/consumer bias because of how they are administered. Third, hospital‐based samples create selection bias since they limit the generalization of the findings only to those women who have used these facilities and may also not account for those who are critically ill or who have transferred from the facility to another institution, leading to an underestimation of the total number of disabled individuals who required C‐sections as a result of their disability and transferred. Finally, the relatively brief length of time during which the study took place limits the number of C‐section admissions that may have been captured by the study, resulting in less specificity of the data. Therefore, all of these limitations should be given due consideration when interpreting the results of this study.

## 5 Conclusion

This study shows that the prevalence of maternal third delay was high. maternal care was not being provided to a number of women in the specified time frame following their arrival at the hospitals. Women being unwanted and unplanned, wanted but unplanned, having no ANC follow‐up, absence of health professionals, Lack of medical supply/medication, and women admitted at daytime were significant predictors of maternal third delay. Strengthen maternal health care services to reduce the third delay. By ensuring admission to hospital in time, ANC registration and follow‐up as required, sufficient health personnel, adequate drugs, and targeted action towards pregnancy of unplanned or unwanted conception. Future studies could benefit from incorporating qualitative approaches and direct observational methods/mixed methods to provide a more in‐depth understanding.

## Author Contributions

**Habtamu Wudu:** conceptualization, methodology, software, data curation, investigation, supervision, writing – review and editing, writing – original draft, formal analysis, project administration, validation, visualization. **Werkneh Minalu:** methodology, writing – original draft, writing – review and editing, visualization, validation.

## Funding

The authors received no financial support for the study design, data collection, analysis, interpretation, or report writing; nor did they have any impact on whether to submit a manuscript for publication.

## Conflicts of Interest

The authors declare no conflicts of interest.

## Transparency Statement

The lead author, Habtamu Wudu, affirms that this manuscript is an honest, accurate, and transparent account of the study being reported; that no important aspects of the study have been omitted; and that any discrepancies from the study as planned (and, if relevant, registered) have been explained.

## Data Availability

The data that support the findings of this study are available from the corresponding author upon reasonable request. The data are not publicly available due to privacy or ethical restrictions.
